# Ventricular interdependence in critically ill patients: from physiology to bedside

**DOI:** 10.3389/fphys.2023.1232340

**Published:** 2023-08-08

**Authors:** Matthieu Petit, Antoine Vieillard-Baron

**Affiliations:** ^1^ Medical Intensive Care Unit, Ambroise Paré Hospital, Assistance Publique–Hôpitaux de Paris, Boulogne-Billancourt, France; ^2^ Inserm, CESP, Paris-Saclay University, Université de Versailles Saint-Quentin-en-Yvelines, Villejuif, France

**Keywords:** ventricular interdependence, hemodynamics, ventricular function, right ventricle (RV), cor pulmonale

## Abstract

The review focuses on the mechanism of ventricular interdependence, a frequently encountered phenomena, especially in critically ill patients. It is explained by the anatomy of the heart, with two ventricles sharing a common wall, the septum, and nested in an acutely inextensible envelope, the pericardium. In pathological situation, it results in abnormal movements of the interventricular septum driven by respiration, leading to abnormal filling of one or the other ventricle. Ventricular interdependence has several clinical applications and explains some situations of hemodynamic impairment, especially in situations of cardiac tamponade, severe acute asthma, right ventricular (RV) overload, or more simply, in case of positive pressure ventilation with underlying acute pulmonary hypertension. Ventricular interdependence can be monitored with pulmonary arterial catheter or echocardiography. Knowledge of this phenomena has very concrete clinical applications in the management of filling or in the prevention or treatment of RV overload.

## Introduction

Besides that both ventricles interact in series and so that the right ventricle is responsible for the filling of the left ventricle by its ejection of blood into the pulmonary circulation, a phenomenon called heart-lung interaction (Fessler, 1997), the performance of one ventricle directly influences the performance of the other, a phenomenon also called ventricular interdependence (Friedberg and Redington, 2014). Ventricular interdependence is especially supported by two mechanisms; the existence of an acutely inextensible envelope, the pericardium, that contains both ventricles leads, in pathological conditions where the pericardial pressure is elevated, to the compression of one ventricle by the other; right and left ventricles share myocardial fibers that directly surround continuously both ventricles, and a common wall, the interventricular septum (IVS).

Ventricular interdependence occurs in most cases in critically ill patients when the pericardial pressure is abnormally elevated, either because of some abnormal liquid inside or because right ventricular (RV) overloading occurs. Due to the sharing of myocardial fibers, a systolic alteration of one can also alter the performance of the other. In this review, our objectives are to physiologically describe abnormal ventricular interdependence and to illustrate clinical applications of such a description.

### Historical considerations

Ventricular interdependence has probably been described for the first time in 1910, when Bernheim (Bernheim, PI, 1910) suggested in ten necropsies of patients with signs and symptoms of RV failure and left ventricular (LV) dilatation that they disclosed a thick LV free wall and ventricular septum with the latter bulging into the RV cavity, provoking a “stenosis of the RV”. In other words, this LV dilatation could impair RV function because of the IVS displacement into the right ventricle, causing venous congestion and congestive cardiac failure. Although this assertion is probably rare, and may even be a “myth”, as Chung et al. did not found any pressure gradient between RV and the pulmonary trunk using pulmonary arterial catheter (Chung et al., 2013), he was the first to describe ventricular interdependence, the septal shift being central in the physiology of this phenomenon. Later, Henderson and Prince (Henderson and Prince, 1914) confirmed the existence of ventricular interdependence in isolated heart of cat, observing that the output of one ventricle decreased when the filling pressure of the other increased. They hypothesized that an IVS shift during diastole explained their findings. More recently, in the past four decades and with the spread of critical care echocardiography (CCE), many studies (Jardin et al., 1981; Vieillard-Baron et al., 1999) demonstrated that IVS position is influenced by diastolic and systolic events and affects the performance of both ventricles.

### Why is there a ventricular interdependence?

It is the anatomy of the heart that underlies ventricular interdependence. Ventricles may be considered as a three-piece system, sharing myocardial fibers and working together, the RV free wall, the IVS and the LV free wall. In experimental studies, Starr et al. (1943), and then Augustus and Bakos showed that despite the complete inactivation of the RV myocardium, the right ventricle was able to maintain a normal pressure in the pulmonary circulation and to develop a normal function only thanks to the energy transmitted by the contraction of the left portion of the fibers common to the two ventricles (Bakos, 1950). These results were confirmed by two studies (Kagan, 1952; Donald and Essex, 1954) where destruction of RV free wall had little impact on peripheral venous pressure or ability to exercise, but need to be tempered by the experience of Fontan and Baudet. (1971), who connected directly the vena cava to the pulmonary circulation in patients with tricuspid atresia. In the latter situation, adapted mean systemic filling pressure drives venous return through the pulmonary circulation without direct LV support. In either cases, this is made possible because in physiological situation, the right ventricle operates on low pressure circulation and can therefore behave like a passive conduit without almost any isovolumetric contraction or relaxation (Redington et al., 1990). Above all, the right and left ventricles share a common wall, the interventricular septum. An increased distension of either ventricle in diastole or systole has been shown to alter the compliance and geometry of the opposite one (Taylor et al., 1967). In physiological conditions, the IVS is oriented so that it is concave to the left ventricle, the pressure into the left ventricle being always higher than the pressure into the right ventricle in diastole and in systole. However, in certain abnormal conditions, it can move to the right or to the left in consequences of variations of trans-septal pressure: if the pressure is higher in the right than in the left ventricle, the IVS will move towards the LV. A diastolic ventricular interdependence has been reported experimentally on *post mortem* and *ex vivo* hearts of dog where progressive RV filling led to progressive abnormal LV diastolic function (Laks et al., 1967; Taylor et al., 1967); as the RV volume and pressure increased, the LV pressure-volume curve were shifted to the left and became steeper. This phenomenon was also described conversely for a rise in LV volume which resulted in an augmentation of RV diastolic pressure (Naeije and Badagliacca, 2017). Then, mechanism of interdependence includes diastolic alteration in ventricular configuration caused by contralateral ventricular volume changes: increasing RV volume (and then pressure) shifts the septum towards the LV cavity and causes a decrease in septal to lateral wall LV dimensions (Bemis et al., 1974; Santamore et al., 1976; Glantz et al., 1978; Jardin et al., 1981). This interdependence may also occur at end-systole when RV is pressure overloaded. In this situation, inverted trans-septal pressure occurs when RV is still ejecting against and obstruction (the ejection time is prolonged) while the left ventricle already starts to relax (Jardin et al., 1981). In all cases, interventricular interdependence occurs when the pericardium is intact (Janicki and Weber, 1980; Spadaro et al., 1981). Pericardial pressure, when abnormally rising because of an increase in the volume or pressure of one of the ventricles or abrupt occurrence of pericardial effusion, magnifies ventricular independence. In an experimental model of RV infarction, Goldstein. (2002) reported that dilation of this ventricle was done to the detriment of the left ventricle by IVS displacement, whereas the preliminary opening of the pericardium allowed the right ventricle to more dilate without harmful effect on the left ventricle, allowing to maintain cardiac output. Furthermore, Glantz et al. (1978) instrumented *in vivo* hearts of dog to study the relationship between LV and RV diastolic pressures with a closed or opened pericardium. With a closed pericardium, RV pressure was is the most powerful predictor of elevated LV diastolic pressure. This situation reversed after the opening the pericardium: LV volume now became the major determinant of LV diastolic with no or poor influence of elevated RV pressure.

### Description and impact of ventricular interdependence in various clinical situations

Very schematically, the IVS shift towards the right ventricle will have harmful consequences on the systemic venous return via the decrease in the pressure gradient for systemic venous return (Guyton et al., 1957) and will therefore lead to decrease in RV stroke volume (SV) and to systemic venous congestion. The IVS displacement towards the left ventricle will lead to reduction in pulmonary venous return and therefore in LV filling, with the consequence of a reduction in LVSV. In this situation, pulmonary congestion is usually not observed because this IVS shift is related to RV overload and impaired RV systolic function, which by itself limits the amount of blood into the pulmonary circulation (ventricular interaction phenomenon).

#### During spontaneous breathing

During “normal” inspiration, RV end-diastolic-volume and stroke volume slightly increase, via an augmentation in the systemic venous return due to a more negative intra-thoracic pressure, and LVSV remains constant or slightly decreases. When inspiration is deep, as in situations of respiratory failure, LVSV always decreases (Hoffman et al., 1965; Summer et al., 1979): increase in RV end-diastolic volume produces a IVS shit toward the LV, causing a decrease in LV compliance and in LV end-diastolic volume. This ventricular interdependence, combined with an increase in LV afterload due to an increase in LV transmural pressure (Buda et al., 1979; McGregor, 1979), ends in a reduction of LVSV (Table 1).

**TABLE 1:** Ventricular interdependence and its consequences on stroke volume of each ventricle in various clinical situations.

	Inspiration		Expiration		Therapeutic interventions
	SV (RV)	SV (LV)	SV (RV)	SV (LV)	
Cardiac tamponade^a^	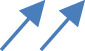	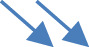	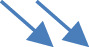	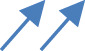	- Limit fluid expansion and avoid positive pressure ventilation
					- Urgent pericardial drainage
Acute Asthma^a^	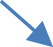	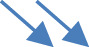	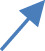	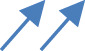	- If MV needed, limit transpulmonary pressure (low tidal volume, low respiratory rate)
					- Use bronchodilatators
Deep spontaneous inspiratory efforts	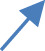	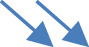	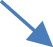	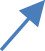	Reduce work of breathing
					- Use oxygen
					- Discuss mechanical ventilation and sedation
Positive pressure ventilation^b^	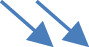	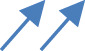	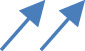	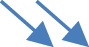	Reduce RV overload
					- Optimize PEEP and driving pressure
					- Limit hypercapnia, improve oxygenation
					- Use prone positionning in case of ARDS, and in severe cases, discuss NO inhalation

SV, stroke volume; RV, right ventricle; LV, left ventricle; PEEP, Positive end-expiratory pressure; ARDS, acute respiratory distress syndrome; NO, nitric oxyde; MV, mechanical ventilation.

^a^ Pulsus paradoxus situation.

^b^ Reverse pulsus paradoxus situation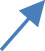 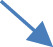
mean that SV increases or decreases, respectively.

#### Cardiac tamponade

**FIGURE 1:**
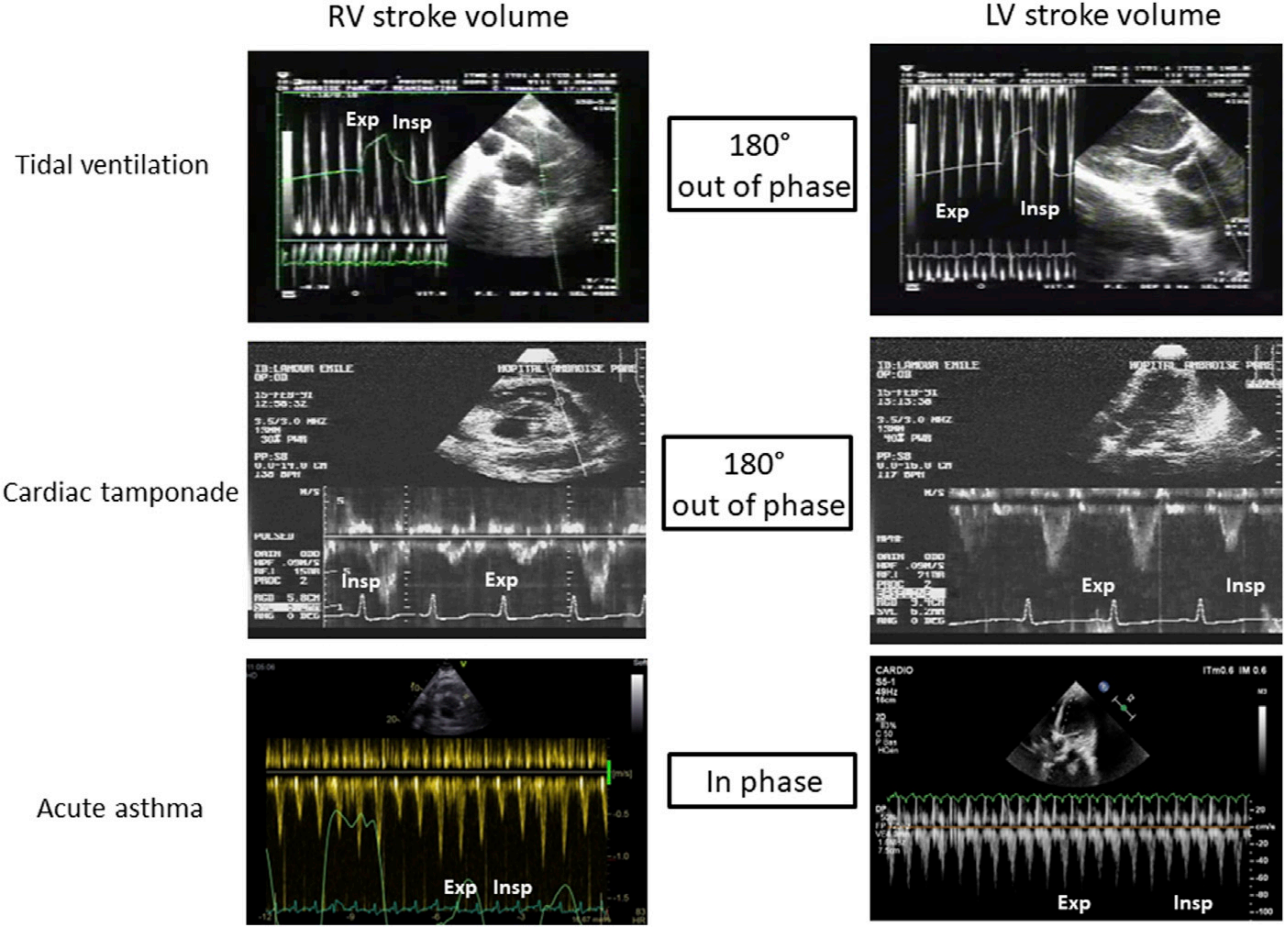
Illustration of ventricular interdependence during tidal ventilation, cardiac tamponade and acute asthma. RV: Righ ventricle, LV: Left ventricle, Exp: expiration, Insp: Inspiration.

It is probably the best known and most evident clinical situation of ventricular interdependence. In physiological conditions, pericardial pressure is negative, close to the pleural pressure. During spontaneous breathing, this pressure decreases at inspiration (more negative), and increases at expiration (less negative). During cardiac tamponade, ventricular interdependence causes pulsus paradoxus (McGregor, 1979; Hamzaoui et al., 2013). Indeed, pericardial pressure increases and may reach up to 15–20 mmHg (Bodson et al., 2011), leading to equalization of cardiac pressures and collapse in systemic venous return (Shabetai et al., 1965). Very early, a competition exists between right atrium (RA) and RV; during systole, pericardial pressure falls as both ventricles eject blood and RA may then be passively filled. Conversely, after ventricular filling during diastole, the ensuing rise in pericardial pressure causes RA compression, a situation often called “pre-tamponade”. If pericardial pressure continues to increase, hemodynamic deterioration leads to the stage of cardiac tamponade with interventricular competition resulting in RV compression at the end-diastole, the left ventricle being filled at the expense of the right, which is the ventricle with the smallest elastance (Bodson et al., 2011). To adapt to this situation, patients develops tachypnea: in inspiration, pleural pressure becomes more negative, is transmitted to the pericardium, which becomes less positive allowing improvement in systemic venous return with a better filling of the right ventricle which increases its stroke volume; however, as a consequence, the IVS is then shifted toward the left ventricle now compressing it and causing a fall in LV filling and LVSV. During expiration, collapsed right ventricle no longer exercises this constraint on the left ventricle and the IVS is then now shifted to the right, especially as previously accumulated blood into the pulmonary circulation now fills the left chambers (pulmonary venous return is much improved) and LVSV increases. The two ventricles are then “180° out of phase”: when the RVSV rises, the LV is compressed, and the LVSV decreases, and *vice versa* (Table 1; Figure 1).

#### Acute asthma

Pulsus paradoxus and ventricular interdependence are also described in severe asthma (Jardin et al., 1982). However, conversely to cardiac tamponade, the two ventricles are “in phase”: the SV of both ventricles decreases at inspiration, and increases at expiration. During inspiration, pleural pressure severely drops (up to −30 mmHg in some cases) from the positive end-expiratory pressure provoked by limitation of expiration. This sudden drop has to effect an increase in RV afterload due to a significant increase in transpulmonary pressure (the lung is severely overinflated other functional residual capacity and extra-alveolar are compressed) and a boost of extra thoracic blood into the right ventricle (increased RV preload) (Guyton et al., 1957). The net result, however, is a decrease in RVSV with a severe RV dilatation, which induces a subsequent leftward IVS shift causing reduction of LV filling and decrease in LVSV (Table 1; Figure 1). Decreased LVSV is also worsened by a drop in transmural LV pressure inducing an augmentation of LV afterload (Stalcup and Mellins, 1977; Buda et al., 1979; Lemaire et al., 1988). During expiration, transpulmonary pressure decreases, unloading the right ventricle, which now ejects a larger stroke volume. This allows the IVS to move back to its normal shape and the left ventricle to be better filled and to better ejects.

Interestingly, while cardiac tamponade and acute asthma both generate pulsus paradoxus, different hemodynamic effects occurs in part explain by ventricular interdependence.

#### Right ventricular overload

Ventricular interdependence has been also reported when the right ventricle is overloaded in pressure or in volume. RV volume overload can result from atrial septal defect, tricuspid insufficiency, or pulmonary insufficiency. It causes an increase in RV end-systolic and end-diastolic volumes with normal RV ejection fraction (Levin et al., 1975; Mathew et al., 1976) but abnormal LV systolic dysfunction through a change in the IVS curvature (Weyman et al., 1976). Usually, the septal shift is limited to the diastole as in systole pressure into the LV remains higher than pressure into the RV (Ryan et al., 1985).

Cor pulmonale is related to pulmonary hypertension, and then reflects RV pressure overload. It can be either chronic with decompensation in the end stage of pulmonary (arterial) hypertension (Champion et al., 2009), or acute occurring on a previously normal right ventricle, such as observed in pulmonary embolism or in acute respiratory distress syndrome (ARDS) where pulmonary hypertension is part of the disease (Price et al., 2012). In both cases, the left ventricle will be crushed by the leftward IVS displacement at end-systole as explained above, which largely explains shock. This inversion of the trans-septal pressure gradient has been measured experimentally in *ex-vivo* heats of cats with each ventricles connected to an independent pump able to gradually generate an augmentation of pulmonary arterial resistance mimicking an obstruction to RV ejection (Elzinga et al., 1980). It has also been reported in humans, using right and left catheterization of patients ventilated for ARDS and submitted to high positive end-expiratory pressure (PEEP) where increasing PEEP until 20 cmH_2_O induced RV dilatation and inverted trans-septal pressure (Jardin et al., 1981).

#### Positive pressure mechanical ventilation

Invasive mechanical ventilation can generate what is called “reverse pulsus paradoxus”, namely, a rise of the arterial systolic and diastolic pressures, presumably related to an inspiratory increase (during insufflation) in left ventricular output (Massumi et al., 1973; Vieillard-Baron and Jardin, 2003). Ventricular interdependence is especially pathological when mechanical ventilation is applied in ARDS. In this situation, tidal ventilation can lead to an increase in RV overload, associating RV dilation, reduction in RVSV, and the IVS displacement towards the left ventricle due to the increase in pericardial pressure. However, this effect on the left ventricle is compensated since tidal ventilation also boosts blood from the pulmonary venous circulation towards the left atrium (secondary to a rise in transpulmonary pressure and a chase effect of blood contained in peri-alveolar vessels), inducing increase in LV filling and in LVSV (Table1; Figure 1), and an fall in LV afterload due to a decrease in LV transmural pressure. The two ventricles are therefore “180° out of phase” (Vieillard-Baron et al., 1999). To note, reverse pulsus paradoxus was also reported in idioventricular rhythm and severe hypertrophic cardiomyopathy (Massumi et al., 1973).

While pulsus paradoxus or reverse pulsus paradoxus are mainly mediated by ventricular interaction (both ventricles in series), ventricular interdependence may either magnify (usually in spontaneously breathing patients) or attenuate (usually in mechanically ventilated patients thanks to its “boosting effect”) such a phenomenon according to the different clinical situations described above.

### Clinical implications of ventricular interdependence in critically ill patients

#### For hemodynamic monitoring

Consequences of ventricular interdependence in critically ill patients are numerous, notably in the field of hemodynamic monitoring. Historically, it has been recommended to monitor hemodynamically unstable patients during septic shock or ARDS with pulmonary arterial catheter (PAC). PAC gives several useful information to manage such patients, like cardiac output, pulmonary capillary wedge pressure (PCWP), pulmonary arterial pressure, central venous pressure. However, its insertion is not without any risk, and more interestingly, PAC does not well appreciate ventricular interdependence. As an illustration, increasing PEEP was suspected to induce LV systolic dysfunction on five patients monitored with both the PAC and left atrial catheter, as cardiac output decreased at high level of PEEP and PCWP and left atrial pressure increased, mimicking LV failure (Lozman, 1974), while we know this is exactly the opposite. This misinterpretation was linked to the effects of high PEEP on RV function, leading to RV overload which shifts the IVS toward the left ventricle, as demonstrated a few years later using echocardiography (Jardin et al., 1981). Same kind of observation was reported in acute asthma where PCWP was unable to detect the underfilled LV during inspiration (Jardin et al., 1982). This is why Doppler echocardiography is perfectly suited to take into account ventricular interdependence, visualizing the respective size of both ventricles as well as the septal motion during respiration, and to monitor RV and LV ejection and their variations.

Eventually, biomarkers like atrial natriuretic peptide can suggest RV overload (Gallo et al., 2023; Volpe et al., 2023).

#### Therapeutic implications

Limiting pericardial pressure is the general principle to correct or limit pathological ventricular interdependence. Beyond compressive pericardial effusion, which must be drained urgently, RV overload, especially in pressure in critically ill patients submitted to mechanical ventilation, is also a factor of increased pericardial pressure and of pathological interdependence as described above. Then, RV overload must be tracked and treated.

In ARDS, a situation where lung compliance is decreased, positive pressure ventilation abnormally increases transpulmonary pressure during tidal ventilation and sometimes during expiration when a too high PEEP is applied (Jardin et al., 1981). As a consequence, pulmonary capillaries are stretched and their caliber reduced, resulting in increase in pulmonary vascular resistance (PVR) (Whittenberger et al., 1960; West et al., 1964). Cyclic increase in PVR during tidal ventilation is responsible for cyclic changes in RV afterload, and then in RV outflow (Vieillard-Baron et al., 1999), eventually leading to reverse pulsus paradoxus as describe above. All factors classically described as being able to increase RV overload in ARDS (Mekontso Dessap et al., 2016) must be taken into account in order to apply a strategy of RV protection if one wants to limit the harmful hemodynamic effects of ventricular interdependence (Paternot et al., 2016). Inhaled NO has also shown its potential benefit to unload the right ventricle, allowing hemodynamic improvement (Puybasset et al., 1994), although its effect on outcome remains uncertain (Gebistorf et al., 2016). At the extreme, veno-venous Extracorporeal Membrane Oxygenation (ECMO) in situation of ARDS with RV failure could correct the detrimental effects of ventricular interdependence by easily controlling blood oxygenation and decarboxylation, two of the major factors of RV overload, and promoting ultra-protective ventilation (Petit et al., 2021; Levy et al., 2022).

Ventricular interdependence also explains the possible deleterious effects of fluid infusion in RV overload. A useless fluid expansion will magnify ventricular interdependence with their deleterious consequences on the left ventricle and hemodynamics. Moreover, an inadequate and massive volume expansion can lead to RV failure by itself (Patterson and Starling, 1914). This deleterious effect of fluid expansion has been described in a few experimental studies. In a model of acute pulmonary circulation obstruction mimicking pulmonary embolism, Ghignone et al. (1984) showed that volume expansion was responsible for supplementary rise in RV pressure and decrease in cardiac output. In this study, the perfusion of norepinephrine was able, by restoring arterial pressure, to reduce RV overload, decrease ventricular interdependence and increase cardiac output. Similar results were found by Angle et al. (1989), and experts recommend to avoid fluid expansion in situation of severe cor pulmonale, i.e., when the right ventricle is already bigger than the left ventricle (Teboul, 2005). To go further afield, in these situations of RV failure and with the knowledge of ventricular interdependence mechanisms, ones can suggest the possible benefit of depletion, either with diuretics or with continuous renal replacement therapy and ultrafiltration. The right ventricle physiologically working in the flat portion of the Starling systolic function curve, no detrimental effect on cardiac output is expected. Conversely, the decline in RV pressure and in RV volume partially corrects the trans-septal pressure gradient, and could improve LV filling and increase cardiac output. This hypothesis has been evaluated in a French randomized controlled trial (Lim et al., 2022) in which the investigators tested the effect of diuretic in intermediate-risk acute pulmonary embolism. They found patients receiving diuretics had better kidney function at day 1 compared to the usual care, suggesting a potentially less degree of venous congestion. Nevertheless, no effect on mortality was reported, probably because of the small number of patients included in the study.

Finally, in acute cor pulmonale with severe circulatory failure, ones can discuss the use of temporary RV extra corporeal support. Peripheral veno-arterial ECMO, by unloading the RV and then, inverting the trans-septal gradient, has been described efficient in situation of high risk pulmonary embolism (Assouline et al., 2022), while same result was suggested using VV ECMO in ARDS (Miranda et al., 2015). Temporary RV assist devices have even been developed, such as double-lumen cannula placed percutaneously (Wang et al., 2015) and advanced through the superior vena cava, the right atrium, the right ventricle and ending in the pulmonary artery. Studies to evaluate these devices are however still needed, but it could be useful in case of severe acute cor pulmonale with high deleterious ventricular interdependence by helping the right ventricle to struggle against an acute rise of pulmonary vascular resistance, situations encountered, for example, in ARDS or in acute chest syndrome due to sickle cell disease.

## Conclusion

Many clinical situations are responsible for pathological ventricular interdependence with adverse effects on hemodynamics. This involves an abnormal increase in pericardial pressure, whatever the cause, and therefore abnormal movements of the interventricular septum towards one ventricle or the other according to changes in the trans-septal pressure gradient. Knowledge of these phenomena has very concrete clinical applications in the management of filling or in the prevention or treatment of RV overload.
